# The Effect of Repetitive Transcranial Magnetic Stimulation on Postural Stability After Acute Stroke: A Clinical Trial

**DOI:** 10.18869/nirp.bcn.8.5.405

**Published:** 2017

**Authors:** Bijan Forogh, Tannaz Ahadi, Maryam Nazari, Simin Sajadi, Lydia Abdul Latif, Seyed Majid Akhavan Hejazi, Gholamreza Raissi

**Affiliations:** 1. Neuromusculoskeletal Research Center, Firoozgar Hospital, Tehran, Iran.; 2. Department of Rehabilitation Medicine, Faculty of Medicine, University of Malaya, Kuala Lumpur, Malaysia.

**Keywords:** Stroke, Transcranial magnetic stimulation, Stroke rehabilitation, Postural balance, Proprioception

## Abstract

**Introduction:**

Balance impairment is a common problem and a major cause of motor disability after stroke. Therefore, this study aimed to investigate whether low-frequency repetitive Transcranial Magnetic Stimulation (rTMS) improves the postural balance problems in stroke patients.

**Methods:**

This randomized double blind clinical trial with 12 weeks follow-up was conducted on stroke patients. Treatment was carried with 1 Hz rTMS in contralateral brain hemisphere over the primary motor area for 20 minutes (1200 pulses) for 5 consecutive days. Static postural stability, Medical Research Council (MRC), Berg Balance Scale (BBS), and Fugl-Meyer assessments were evaluated immediately, 3 weeks and 12 weeks after intervention.

**Results:**

A total of 26 patients were enrolled (age range=53 to 79 years; 61.5% were male) in this study. Administering rTMS produced a significant recovery based on BBS (df=86, 7; F=7.4; P=0.01), Fugl-Meyer Scale (df=86, 7; F=8.7; P<0.001), MRC score (df=87, 7; F=2.9; P=0.01), and static postural stability (df=87, 7; F=9.8; P<0.001) during the 12 weeks follow-up.

**Conclusion:**

According to the findings, rTMS as an adjuvant therapy may improve the static postural stability, falling risk, coordination, motor recovery, and muscle strength in patients with stroke.

## 1. Introduction

Stroke as the most disabling neurologic injury and third leading cause of death, is one of the most important challenges of health systems. It significantly reduces the patients’ quality of life and can also increase the health care costs. The annual incidence of this disease is very high. According to the World Health Organization, 37 million cases of stroke were estimated (Mathers, Fat, & Boerma, 2008) in 2004. Although the incidence of stroke decreased in developed countries since the early 1970s, this trend was reversed in developing countries (Feigin, Lawes, Bennett, Barker-Collo, & Parag, 2009). Balance and postural stability impairment is a common problem and a major cause of motor disability (locomotion) after stroke (Lee, 1989; Rode, Tiliket, & Boisson, 1997). Today, several tools and techniques are used to improve post-stroke postural balance and performance. Among them, repetitive transcranial magnetic stimulation (rTMS) has been used as an adjuvant therapy (Dimyan & Cohen, 2010).

The first successful rTMS study was performed in 1985 by Anthony Barker and his colleagues in England (Corthout, Barker, & Cowey, 2001). It is a simple and non-invasive procedure that can have positive effects on motor recovery in post-stroke hemiparesis (Dimyan & Cohen, 2010; Weiduschat et al., 2011). This procedure has been used in two methods: low-frequency stimulation (≤1 Hz) to decrease the excitability of the unaffected brain hemisphere or high-frequency stimulation (>1 Hz) to increase excitability of the affected brain hemisphere (Forogh, Yazdi-Bahri, Ahadi, Fereshtehnejad, & Raissi, 2014; Hao, Wang, Zeng, & Liu, 2013; Khedr, Abdel-Fadeil, Farghali, & Qaid, 2009; Khedr, Etraby, Hemeda, Nasef, & Razek, 2010). The effect of rTMS on balance is still unclear. Since rTMS may improve patient’s motor recovery, it may have a positive effect on functional balance in the subacute and chronic phase of stroke. Therefore, this study aimed to investigate whether low-frequency rTMS improves the balance problems in stroke patients.

## 2. Methods

### 2.1. Study design and setting

This randomized double blind clinical trial with 12 weeks follow-up was conducted in Firoozgar hospital, Tehran, Iran between April to December 2014. Patients were selected con-secutively from those who were admitted at Firoozgar Center of Physical Medicine and Rehabilitation. The protocol of the study was approved by the Ethics Committee of Iran University of Medical Sciences. Signed informed consent was attained from all patients prior to enrollment in the study.

### 2.2. Subjects

Inclusion criteria were as follows: stroke patient with subacute and chronic ischemic and hemiplegic stroke documented by Computed Tomography (CT) or MRI; at least one month has elapsed from stroke; first-ever cerebral infarction; ability to perform 3-step command (3 points); no cognitive impairment, impaired patient’s balance and gait; the ability to walk with or without support; and with Functional Ambulation Categories more than one.

Patients were not included in the study if they had: a second stroke, bilateral weakness; the cerebellum or brain stem involvement, proprioception impairment, hemianopsia or another visual impairment, vestibular dysfunction, neurologic comorbidity other than stroke like neuropathy, severe postural instability, orthopedic problems, significant cognitive problem, receptive aphasia, epilepsy or seizures after stroke, and pathological conditions referred as contraindications of rTMS (presence of a metallic implant inside the eye or the brain, the external fixator, cardiac pacemaker). For sample size calculation, according to Emara et al., (2010) study, with considering the type I error equal to 5% (α=0.05) and an accuracy of 1% (d=0.01), the number of patients required in each group was found as 10. Randomization was done by an independent researcher. Subjects were randomly assigned to two groups; rTMS and Sham group.

### 2.3. Intervention

**Figure 1.**
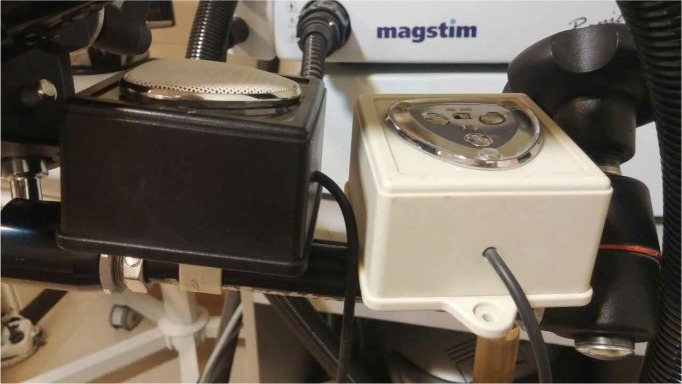
The device was installed for Sham stimulation.

Treatment was carried in 5 consecutive days, with 1 Hz rTMS in contralateral brain hemisphere over the primary motor area for 20 minutes (1200 pulses), in sitting position. Low-frequency rTMS was administered by a 70-mm figure-8 coil connected to Magstim R30 stimulator (MagVenture, Denmark). The optimal site and intensity of stimulation was deter-mined based on proposed method of Kondo et al., (2013). As in the real rTMS group, for Sham stimulation, we recorded the sound of stimulator. A small speaker was installed on the stimulation coil handle. The coil was placed on the head, adjustments were done on the rTMS monitor, but speaker was activated by a switch behind the patient. A sound mimicking the real rTMS was played for the patient (Figure 1).

### 2.4. Measurement

Clinical and postural evaluations were performed prior to the first session, immediately after the rTMS course, 3 weeks and then 3 months later. Static postural stability was assessed by a balance assessment system (Biodex, Balance System SD, 115 VAC, Germany). For static postural stability, the patients’ standing on a stable flat were evaluated. The patient’s legs were placed on 30-degree angle for 20 to 60 seconds and they were asked to maintain their standing balance. Balance function without external perturbation and the open as well as closed eyes was evaluated. Medical Research Council (MRC) scale was used to assess muscle strength (Paternostro-Sluga et al., 2008). This scale is a six grade scoring system in which 0 denotes no movement and 5 normal power. Static and dynamic balance ability was evaluated by Berg Balance Scale (BBS) (Steffen, Hacker, & Mollinger, 2002). It comprises 14 items and each item is scored from 0 (unable) to 4 (independent) with maximum total score of 56. Finally, Fugl-Meyer assessment was used to assess motor recovery after stroke. It is scored on a 3-point ordinal scale (0–2) with maximum of 226 (Atler, Malcolm, & Greife, 2015).

### 2.5. Data analysis

SPSS 22.0 was used for statistical analysis. Descriptive and inferential statistics including repeated measures ANOVA to assess trends of improvement within each group, 2-way ANOVA for detection of statistical difference of recovery between two groups over the time were used. In all analyzes, P<0.05 was considered as significant.

## 3. Results

A total of 26 patients were enrolled (age range=53 to 79 years; 61.5% were male) in this study. Left brain hemisphere was affected in 18 (69.2%) patients. Duration of the disease in 22 (84.6%) was more than 6 months. During 12 weeks follow-up, 11 patients (5 patients in the treatment and 6 patients in Sham group) withdrew from the study.

**Figure 2.**
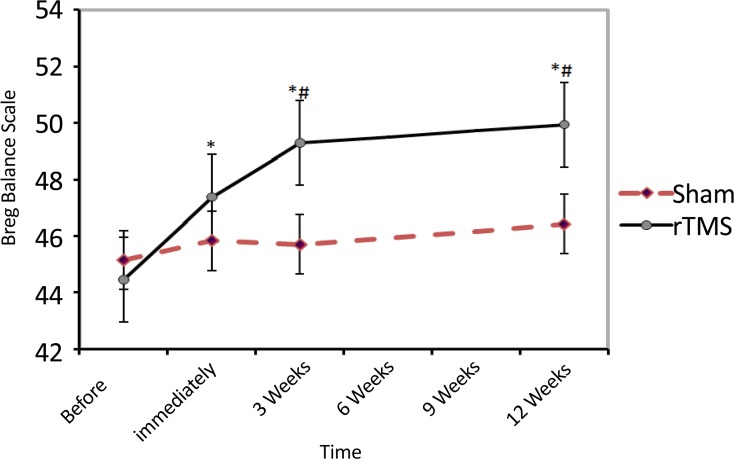
The impact of rTMS on functional balance during 12 weeks follow-up of patients, based on the Berg Balance Scale * Indicates a significant difference with the pre-intervention period. # Represents a significant difference with the corresponding time in Sham group. No data were collected during 6- and 9-weeks periods.

Administration of rTMS produced a significant recovery in BBS during 12 weeks follow-up (compared with preintervention time). Mean(SD) BBS of rTMS group at baseline was 44.6(5.2), after 5 sessions of rTMS, 3 weeks and 12 weeks later it reached to 47.6(4.4), 49.6(4.4) and 50.1(3.9), respectively (df=3; F=7.5; P=0.004). Compared with Sham group, BBS in patients treated with rTMS after 3 weeks [49.6(4.4) vs. 46(44); P=0.03] and 12 weeks [50.1(3.9) vs. 46.7(5.8); P=0.02]showed a significant increase (df=7, 86; F=7.4; P=0.01) (Figure 2).

**Figure 3.**
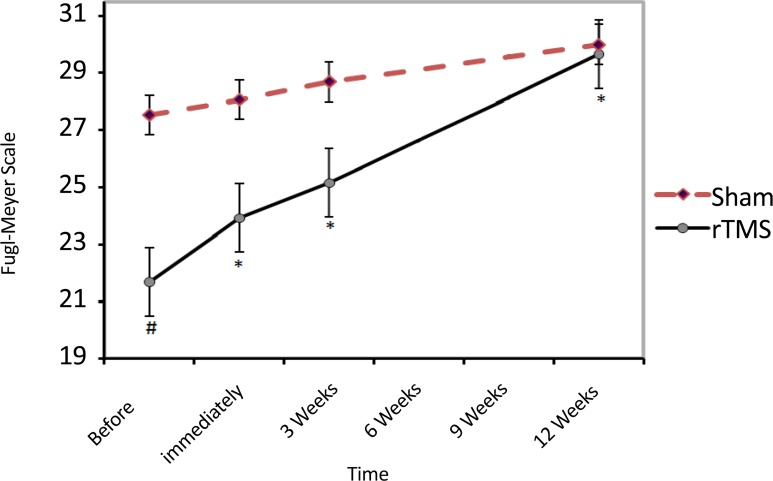
The impact of rTMS on motor recovery during three weeks of follow-up based on the Fugl-Meyer Scale. * Indicates a significant difference with the pre-intervention period. No data were collected during 6- and 9-weeks periods.

Administration of rTMS improved motor recovery after stroke during 12 weeks follow-up (compared with preintervention time). Mean(SD) Fugl-Meyer Scale at baseline in rTMS group was 22.7(6.1). The Mean(SD) score immediately, three weeks and 12 weeks after rTMS, reached to 24.3(4.9), 26.2(4.2) and 28.7(4.2), respectively (df=3; F=15.3; P<0.001). Mean(SD) Baseline Fugl-Meyer score in rTMS group was significantly lower than Sham group (df=86, 7; F=8.7; P<0.001) while 3 months after treatment it became nearly the same score [29.0(2.6) vs. 28.7(4.3); P>0.99)] (Figure 3).

**Figure 4.**
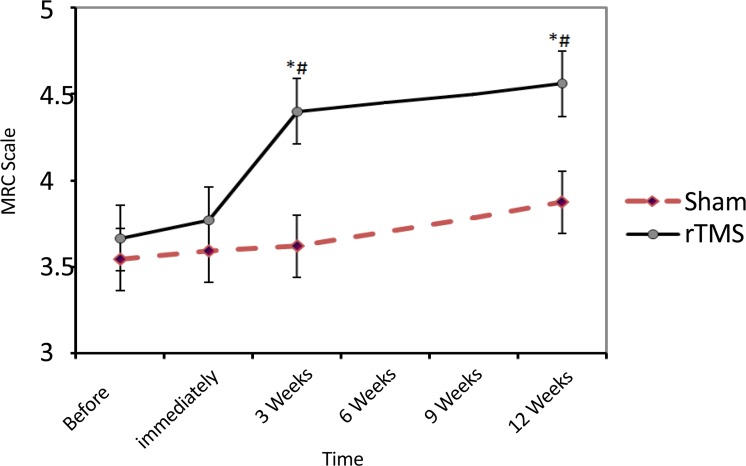
The impact of rTMS on muscle strength during 12 weeks follow-up based on the Medical Research Council (MRC) scale. * Indicates a significant difference with the pre-intervention time. # Represents a significant difference with the corresponding time in the Sham group. No data were collected during 6- and 9-weeks periods.

Treatment with rTMS resulted in significant increase in muscle strength (Figure 4). Although the Mean(SD) MRC score in rTMS group did not change after 5 sessions [3.8(0.8) compared with 3.7(0.9); df=2; F=1.0; P=0.35], three weeks and 12 weeks after treatment it significantly increased to 4.4(0.5) and 4.6(0.5), respectively (df=3, F=13.8; P<0.001). Two-way ANOVA revealed the Mean(SD) MRC in rTMS group compared with Sham significantly improved at the 3 weeks [4.4(0.5) vs. 3.6(0.9); P=0.03] and 12 weeks [3.9(0.8) vs. 4.4(0.5); P=0.04] after treatment (df=7, 87; F=2.9; P=0.01).

**Figure 5.**
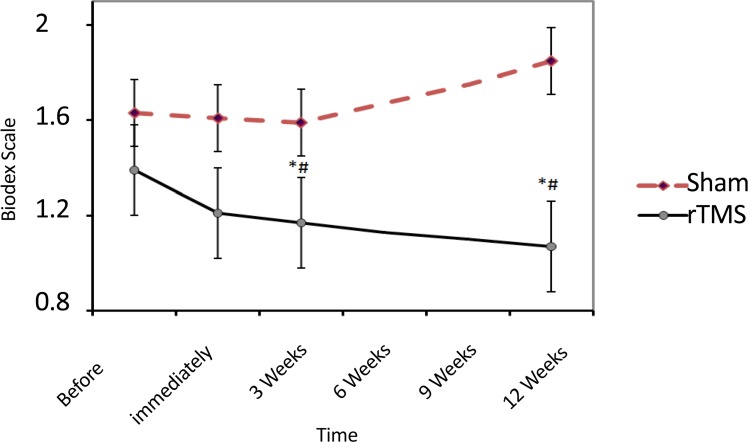
The impact of rTMS on the static postural stability in the 12 weeks follow-up. * Indicates a significant difference with the pre-intervention. #Indicating significant difference with corresponding time in Sham group. No data were collected during 6- and 9-weeks periods.

Static postural stability was improved in rTMS group over 12 weeks (Figure 5). This improvement was significant compared with before intervention (df=3; F=7.7; P<0.00) and corresponding times in Sham (df=7; 87; F=9.8; P<0.001). Mean(SD) MRS scores for the 3 weeks and 12 weeks after rTMS in treatment group were 1.12(0.6) and 1.14(0.6), respectively while in the Sham group they were 1.6(0.6) and 1.85(0.4), respectively.

## 4. Discussion

The findings of this study showed that rTMS as an adjuvant therapy can significantly improve the static postural stability, functional recovery and muscle strength in patients with stroke. To our knowledge, this is the first study on the role of rTMS on balance stability. However, several studies have demonstrated beneficial impact of rTMS on motor recovery after stroke. For example, Khedr et al. showed the beneficial effect of rTMS on stroke related dysphagia and functional recovery (Khedr et al., 2009; Khedr et al., 2010; Khedr et al., 2014). Avenanti et al. concluded that combined time-locked rTMS was an effective and encouraging method for improvement of chronic stroke patients with mild motor impairment (Avenanti, Coccia, Ladavas, Provinciali, & Ceravolo, 2012).

In addition, Corti et al. in their review suggested that rTMS applied to the affected brain hemisphere was a safe method and could be considered as a valid technique for restraining brain function and contributing to motor recovery after stroke (Corti, Patten, & Triggs, 2012). Takeuchi et al. demonstrated that rTMS was a promising non-invasive tool for the hand function recovery (Takeuchi, Chuma, Matsuo, Watanabe, & Ikoma, 2005). Emara and colleagues also showed that rTMS might improve post-stroke functional recovery. These re-searchers reported that the recovery rate in 1 Hz rTMS treated group is better than 5 Hz one (Emara et al., 2010).

The underlying mechanisms of rTMS in stroke recovery have remained unclear. However, the effectiveness of these techniques in the excitability of neurons has been proved (Iyer, Schleper, & Wassermann, 2003). rTMS use electromagnetic induction to produce an electric current across the scalp and skull without any physical contact (Eichhammer, Langguth, Marienhagen, Kleinjung, & Hajak, 2003). Researchers generally believe that rTMS through changing the excitability of the nerve cells such as Long-Term Potentiation (LTP) and Long-Term Depression (LTD) causes an excitatory or inhibitory effect (Speer et al., 2000). Serotonin receptors, noradrenergic and dopaminergic change are also likely to be affected by rTMS (Wassermann & Lisanby, 2001). Brain-Derived Neurotrophic Factor (BDNF) has an essential role in neuronal plasticity (Hashimoto, 2013). For example, release of BDNF after physical exercise may cause considerable modification in structure and function of astrocytes that protects against glutamate toxicity during aging and a number of neurodegen-erative disorders (Fahimi et al., 2016). Recent reports suggest that BDNF mediates, at least in part, the therapeutic effects of rTMS. Chang et al. showed that BDNF gene polymorphism has negative effect on the outcome of rTMS on the motor recovery of upper extremities in stroke patients (Chang et al., 2014).

Niimi et al. showed that the combination of rehabilitation and low-frequency rTMS may improve motor function in the affected limb, by activating brain-derived neurotrophic factor processing (Niimi et al., 2016). In the first week after the stroke, the presence of excitatory potentials in paresis limb in response to stimulation of the affected hemisphere may be a good predictor of functional recovery (Catano, Houa, Caroyer, Ducarne, & Noel, 1995; D’Olhaberriague et al., 1997; Escudero, Sancho, Bautista, Escudero, & López-Trigo, 1998; Hendricks, Pasman, Merx, van Limbeek, & Zwarts, 2003; Rossini et al., 1994; Rossini et al., 1998). On the contrary, the absence of such potentials is associated with poor recovery (Shimizu et al., 2002). In addition, neuroimaging studies show that patients with poor recovery have higher levels of brain activity in unaffected hemisphere (Ward & Frackowiak, 2006). This excitatory imbalance between two hemispheres, decline during the first month after stroke. This period is simultaneously associated with functional improvement (Cicinelli, Traversa, & Rossini, 1997; Delvaux et al., 2003; Traversa, Cicinelli, Pasqualetti, Filippi, & Rossini, 1998).

The reason for using rTMS in stroke patients is based on these changes. It is believed that stroke leads to loss of inhibitory effect of damaged hemisphere on the unaffected side. When inhibition of the normal hemisphere is removed; the excitatory function of this hemisphere increases. Subsequently, inhibitory effect of normal hemisphere on affected hemisphere will be increased. Therefore, the use of low-frequency rTMS over the unaffected hemisphere may decrease inhibitory signals and consequently damaged hemisphere be reactivated, leading to better functional recovery. There are several studies to prove this hypothesis. For example, Mansur et al. first demonstrated that inhibition of the unaffected hemisphere by low-frequency rTMS (1 Hz) led to substantial improvement in limb performance (Mansur et al., 2005). In addition, Takeuchi et al. reported that rTMS of contralesional primary motor cortex improves hand function after stroke (Takeuchi et al., 2005).

The main limitation of this study is its low sample size that could affect the results. However, the minimum power obtained with this sample size was 81%, so this limitation was largely overcome. Another limitation was short follow up period. So, we were not able to determine the long-term effects of rTMS. The present study showed that rTMS as an adjuvant therapy may improve the static postural stability, falling risk, coordination, motor recovery, and muscle strength in patients with stroke. These effects could persist up to 3 months. Further research should be conducted with larger sample size.
